# Characterization of forced localization of disordered weakly coupled micromechanical resonators

**DOI:** 10.1038/micronano.2017.23

**Published:** 2017-07-03

**Authors:** Hemin Zhang, Honglong Chang, Weizheng Yuan

**Affiliations:** 1 Ministry of Education Key Laboratory of Micro and Nano Systems for Aerospace, School of Mechanical Engineering, Northwestern Polytechnical University, Xi’an 710072, China

**Keywords:** anti-resonance, eigenvalue loci veering, energy confinement, forced localization, mode localization, weakly coupled resonators

## Abstract

The mode localization phenomenon of disordered weakly coupled resonators (WCRs) is being used as a novel transduction scheme to further enhance the sensitivity of micromechanical resonant sensors. In this paper, two novel characteristics of mode localization are described. First, we found that the anti-resonance loci behave as a linear function of the stiffness perturbation. The anti-resonance behavior can be regarded as a new manifestation of mode localization in the frequency domain, and mode localization occurs at a deeper level as the anti-resonance approaches closer to the resonance. The anti-resonance loci can be used to identify the symmetry of the WCRs and the locations of the perturbation. Second, by comparing the forced localization responses of the WCRs under both the single-resonator-driven (SRD) scheme and the double-resonator-driven (DRD) scheme, we demonstrated that the DRD scheme extends the linear measurement scale while sacrificing a certain amount of sensitivity. We also demonstrated experimentally that the amplitude ratio-based sensitivity under the DRD scheme is approximately an order of magnitude lower than that under the SRD scheme, that is, the amplitude ratio-based sensitivity is −70.44% (N m^−1^)^−1^ under the DRD scheme, while it is −785.6% (N m^−1^)^−1^ under the SRD scheme. These characteristics of mode localization are valuable for the design and control of WCR-based sensors.

## Introduction

Microelectromechanical systems (MEMS) resonators have been widely used in various applications such as communications^1,2, 3^, medical diagnostics^4,5^, and inertial navigation^6,7,8^. Typically, the structural parameters of the resonant element change with the addition of ambient physical/chemical quantities^9^. Variations of the physical/chemical quantities can then be measured by monitoring the shift of the resonant frequency. To date, most sensing applications are based on the single degree of freedom (SDOF) resonator. Recently, multi-degrees of freedom (MDOF) micromechanical coupled resonators have drawn more attention. Some physicists have used them to explore basic physical phenomena such as synchronization^10,11^ and level repulsion^12^. For the MEMS community, a more interesting application is to use MDOF micromechanical coupled resonators in sensors with ultra-high sensitivity. Researchers have developed strongly coupled resonators using frequency splitting as the output metric to improve the sensitivity by more than 20%^13^. Using weakly coupled resonators (WCRs) and taking the eigenstate or amplitude ratio as the output metric can enhance the sensitivity by more than 2 orders of magnitude^14^. A variety of sensors based on WCRs with different degrees of sensitivity enhancement have been implemented, such as mass sensors^14,15,16,17^, electrometers^18,19^, stiffness sensors^20,21^, accelerometers^22^, and tilt sensors^23^.

Mode localization is the theoretical basis for the sensitivity improvement of WCRs-based sensors. As a manifestation of Anderson localization^24^ in the field of structural dynamics, mode localization^25,26,27,28, 29,30^ has been studied for more than three decades. When mode localization occurs, the WCRs exhibit drastic energy confinement on a specific mode. Energy confinement, which is the essential characteristic of the mode localization, can be described as “the vibrational energy injected into the structure by an external source cannot propagate over arbitrarily large distances but is instead substantially confined to a region close to the source”^27^. Therefore, the energy confinement of the mode localization can be regarded as the spatial redistribution of the vibrational energy.

The eigenvalue loci veering^29^ of the WCRs is another well-known characteristic and describes the phenomenon in which the frequency loci of the vibration modes diverge and do not intersect as they approach one another. Energy confinement and eigenvalue loci veering are two different manifestations of the same phenomenon, and they cover the two important parameters of the coupled systems, i.e., the eigenstates and eigenvalues. These two characteristics of WCRs have been experimentally verified using micromechanical resonators in previous studies^19,31^. In this paper, two additional characteristics of the mode localization of disordered WCRs are explored. It would be helpful to the MEMS community to understand and apply this new sensing mechanism to their sensors.

First, we found and verified that the anti-resonance loci behave as a linear function of the stiffness perturbation. It is strange that the anti-resonance^32^ locations of the disordered WCRs, which play an important role in vibration control and dynamic model updating^33^, have seldom been linked to mode localization in the past, although the anti-resonance indicates a valley in the vibrational magnitude of the frequency domain^34^. Several aspects of the linear anti-resonance behavior are very meaningful. With the anti-resonance approaching a certain mode (the resonance point), the vibrational energy will be confined to the other mode, which qualitatively indicates the occurrence of mode localization. Furthermore, the proportions of vibrational energy in the two modes of the disordered weakly coupled resonators are established to quantitatively characterize the mode localization. Mode localization occurs at a deeper level as the anti-resonance approaches the resonance more closely, and the proportions of vibrational energy can be used to determine the upper and lower limits of the measurement range of the mode-localized sensors. Therefore, the anti-resonance behavior can be regarded as a new and succinct manifestation of mode localization. As an application, the anti-resonance behavior can be used to identify which resonator is perturbed by observing whether the anti-resonance frequency changes.

Second, we found that the amplitude ratio or frequency-based sensitivity under the double-resonator-driven (DRD) scheme is approximately an order of magnitude lower than that under the single-resonator-driven (SRD) scheme, while the linear measurement range under the DRD scheme is extended by a factor of more than three. Under the SRD scheme, a single resonator is directly driven, while under the DRD scheme, both coupled resonators are driven simultaneously by forces with equal magnitude and of the same phase. The DRD scheme is very useful to the MEMS community for sensor design because it is easier to stabilize the resonator in a specific mode than in the SRD scheme.

## Materials and methods

### Responses of disordered WCRs

**Figure 1:**
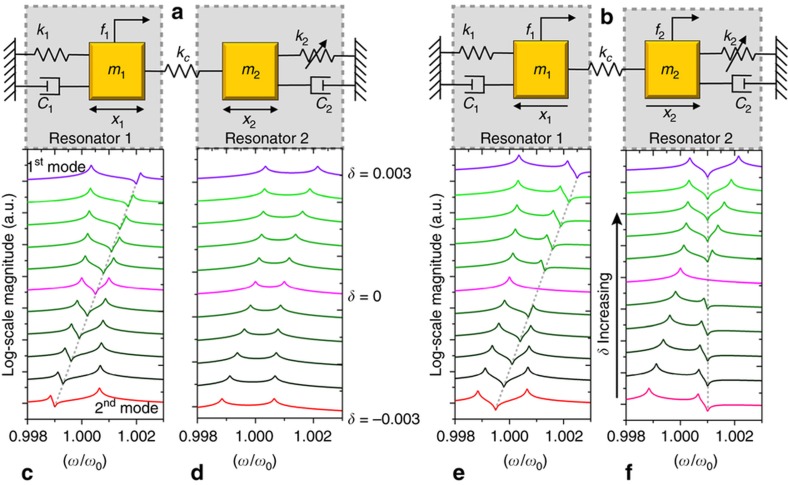
(**a**) Model of disordered weakly coupled resonators under the SRD scheme. (**b**) Model of disordered weakly coupled resonators under the DRD scheme. (**c**) and (**d**) Responses of Resonator 1 and Resonator 2 with different stiffness perturbations under the SRD scheme. (**e** and **f**) Responses of Resonator 1 and Resonator 2 with different stiffness perturbations under the DRD scheme.

In this paper, a 2-DOF WCR system is taken as an example for theoretical modeling. It can be modeled as the linearly coupled chains of two spring-mass-damper systems, as shown in Figure 1a. In the WCRs, the coupling force is much smaller than the elastic force; i.e., *k*_*c*_≪*k*. For two identical coupled resonators (*m*_1_=*m*_2_=*m*, *k*_1_=*k*_2_=*k*) with a small stiffness perturbation *δ* on Resonator 2, the dynamic equations of the model can be written as

(1)d2x1dt2+ωQdx1dt+ω2(1+κ)x1−κω2x2=f1md2x2dt2+ωQdx2dt+ω2(1+κ+δ)x2−κω2x1=f2m}

where *ω* is the frequency of the driving force, *Q* is the quality factor, *κ*=*k*_*c*_/*k* is the coupling factor, *δ*=Δ*k*/*k* is the stiffness perturbation on Resonator 2, *x*_1_ and *x*_2_ are the displacements of the two masses, and *f*_1_ and *f*_2_ are the driving forces applied to the two masses.

In the 2-DoF system, the two vibration modes are the in-phase mode (first mode), in which the two masses move in the same direction, and the out-of-phase mode (second mode), in which the two masses move in opposite directions. The eigenvalues and amplitude ratios of the two modes with no perturbation (Δ*k*=0) are:

(2)ω12=ω02,ω22=(1+2κ)ω02,u1=1,u2=−1

where $\omega_{0}=\sqrt{k/m}$, *ω*_1_ and *ω*_2_ are the resonant frequencies and *u*_1_ and *u*_2_ are the amplitude ratios of the first and second modes. In this paper, a short beam was used to mechanically couple the two resonators. Therefore, the coupling factor *κ* is positive, so that the first mode (in-phase mode) is the lower-order mode, and the second mode (out-of-phase mode) is the higher-order mode.

According to Equation (1), the vibrational displacements of the two masses under the SRD (*f*_1_≠0, *f*_2_=0) and DRD (*f*_1_=*f*_2_=*f*) schemes are, respectively, written as:

(3){XS1∝Λ−1(−(ωω0)2+1+κ+δ+j1Qωω0)XS2∝Λ−1κXD1∝Λ−1(−(ωω0)2+1+2κ+δ+j1Qωω0)XD2∝Λ−1(−(ωω0)2+1+2κ+j1Qωω0)

where subscripts S and D represent the SRD and DRD schemes, respectively, and *Λ* is the denominator of the transfer function, which is written as:

(4)Λ=[(ωω0)4−(2+2κ+δ+1Q2)(ωω0)2+(1+2κ+δ+δκ)−j1Q[2(ωω0)3−(2+2κ+δ)ωω0]]

Under the SRD scheme (Figure 1a), it can be observed that the magnitude of the second mode is much larger than that of the first mode when *δ*<0, which means that the vibrational energy of Resonator 1 is mostly confined to the second mode (Figure 1c) in the frequency domain. When *δ* increases from negative to positive, the energy that was confined to the second mode gradually transfers to the first mode. The energy is equally distributed between the two modes when *δ*=0. When *δ*>0, the vibrational energy is predominantly confined to the first mode. For Resonator 2 (Figure 1d), the vibrational energy is always evenly distributed between the two modes, and the vibrational energy reaches a maximum when *δ*=0. The phenomenon in which the vibration is confined to a specific mode is termed energy confinement^27^ and is always regarded as the essence of mode localization.

Usually, in the MEMS field, the DRD scheme is used to ensure that the coupled resonators vibrate in a certain mode, such as the in-phase mode. We can see from Figures 1e and f that when *δ*=0, the vibrational energy is completely confined to the first mode for both Resonator 1 and Resonator 2. With the variation of *δ*, for Resonator 1, the vibrational energy of the second mode may exceed that of the first mode at a certain value where *δ*<0. This indicates that the structural disorder will make the vibrational energy of the WCRs not ideally locked in a certain mode, even with the DRD scheme.

### Anti-resonances of disordered WCRs

**Figure 2:**
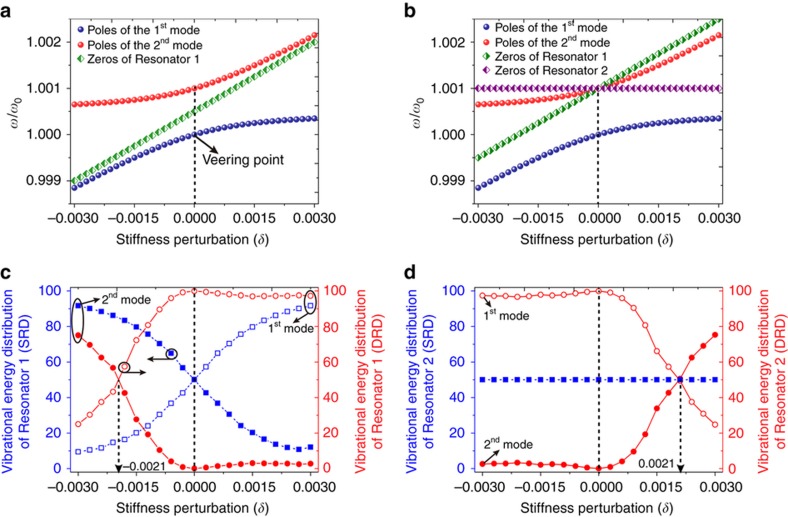
(**a**) Poles and zeroes of the two resonators under the SRD scheme. (**b**) Poles and zeroes of the two resonators under the DRD scheme. (**c**) Vibrational energy distribution of Resonator 1 under the SRD and DRD schemes. (**d**) Vibrational energy distribution of Resonator 2 under the SRD and DRD schemes. Here, the quality factor (*Q*) was set to 10 000 and the coupling factor (*κ*) was set to 0.001.

In the 2-DoF vibration system, the resonances represent the resonant frequencies of the mass-stiffness matrix and the poles of the transfer functions and appear in the frequency domain where the magnitude reaches a maximum. According to Equation (4), the expressions of the poles are

(5)ωi2=(1+κ+12δ∓12δ2+4κ2)ω02,i=1,2

where the subscript *i* represents the *i*th vibration mode. The resonant frequencies of the two modes versus the stiffness perturbation are shown in Figures 2a and b. From Figure 2a, we can see that the poles (resonances) of the two modes repulse quickly when they approach each other at the veering point (the point where the two resonance loci separate from each other). This is the eigenvalue loci veering phenomenon and is regarded as another important manifestation of mode localization.

In classical vibration measurement, it is known that for a 2-DOF system, there will be an anti-resonance between the two vibration peaks^35^. The anti-resonance represents the zero of the transfer function and is always accompanied by a vibrational magnitude valley in the frequency domain. When Resonator 2 is perturbed, zeroes of the two resonators under the two driving schemes as functions of the stiffness perturbation are:

(6)[ZS1ZD1ZS2ZD2]=[1+κ+δω01+2κ+δω0/1+2κω0]

where *Z*_S1_, *Z*_S2_ represent zeros of Resonator 1 and Resonator 2 under the SRD scheme and *Z*_D1_, *Z*_D2_ represent zeros of Resonator 1 and Resonator 2 under the DRD scheme, respectively.

On the basis of Equation (6), we can obtain the anti-resonance behaviors of the coupled resonators as functions of the stiffness perturbation. Under the SRD scheme, there is an anti-resonance for Resonator 1, and there is no anti-resonance for Resonator 2, as the green diamond line shows in Figure 2a, while under the DRD scheme, there are anti-resonances for both Resonator 1 and Resonator 2. The anti-resonance loci of the two resonators both cross with the resonance loci of the second mode. The anti-resonance loci of Resonator 1 vary linearly with the stiffness perturbation, while the anti-resonances of Resonator 2 are always constant.

When Resonator 1 is perturbed, the anti-resonances (zeroes) under the SRD scheme can be similarly derived as

(7)[ZS1′ZD1′ZS2′ZD2′]=[1+κω01+2κω0/1+2κ+δω0]

From Equation (7), it can be concluded that the anti-resonances of Resonator 1 under the SRD scheme are not related to the stiffness perturbation *δ*, which is different from the linear behavior, as shown in Figure 2a. Therefore, the anti-resonance behavior can be used to identify which resonator is perturbed. This is quite useful in the eventual application of mass sensing.

For example, frequencies of the zero points under SRD scheme when Resonator 1 is assumed to be perturbed by an additional mass Δ*m* can be written as:

(8)ZmS1=(k+kc)/m

where *Z*_*m*S1_ means the anti-resonance frequency of Resonator 1 with mass perturbation added to Resonator 1. From Equation (8), it can be concluded that the anti-resonance frequency is independent of the mass perturbation Δ*m*. In contrast, when Resonator 2 is assumed to be perturbed, the derived anti-resonance frequency is:

(9)ZmS1′=(k+kc)/(m+Δm)

where ${Z_{mS1}}^{\prime }$ is the anti-resonance frequency of Resonator 1 with mass perturbation added to Resonator 2. It can be concluded from Equation (9) that the anti-resonance frequency is approximately a linear function of Δ*m* in a small range of Δ*m*≪*m*. Therefore, we can identify which resonator is applied with a mass perturbation by observing whether the anti-resonance frequency changes.

### Quantitative description of localization

To quantitatively describe the level of the mode localization, we calculated the proportions of vibrational energy of the two modes based on Equations (3) and (4). The vibrational energy is based on the potential energy formula $E=\frac{1}{2}mx^{2}$. The quantitative energy distributions of Resonator 1 under the SRD scheme are shown by the blue squares in Figure 2c. The energy proportions of the first and second modes are each 50% at the veering point, but they become 9.32 and 90.68%, respectively, when *δ* decreases to −0.003. The energy distributions in the two modes exchange with each other when *δ* is inverted to 0.003. This indicates that when *δ* is far from zero, the vibrational energy is mostly confined to a particular mode and not distributed between in the two modes. In contrast, for Resonator 2 under the SRD scheme, as shown by the blue squares in Figure 2d, the vibrational energy is always evenly distributed between the two modes, both with and without stiffness perturbation.

Interestingly, for Resonator 1 under the DRD scheme, as shown by the red circles in Figure 2c, it can be observed that the vibrational energy split in the first and second modes is 100 and 0% at the veering point, while an even energy proportion appears at the point where *δ*=−0.0021. For Resonator 2 under the DRD scheme, as shown by the red circles in Figure 2d, an even split appears when *δ*=0.0021. This indicates that the vibrational energy is inclined to be predominantly confined to the first mode when *δ* approaches zero from the even energy proportion point, which is different from the variation trend under the SRD scheme. This can be explained by the fact that the second mode shape ([1, −1]) of the tuned system is ideally orthogonal to the external force vector [1, 1]^T^, which makes the magnitude of the second mode 0 at the veering point. With an increase of the stiffness perturbation, the eigenvector of the second mode is no longer equal to the ideal value ([1, −1]) because the amplitude ratio varies with the change in the stiffness perturbation: $u_{2}=\left(-\delta -\sqrt{\delta^{2}+4\kappa^{2}}\right)/2\kappa$. Thus, the eigenvector of the second mode will no longer be orthogonal to the external force vector, and the energy proportion of the second mode would therefore not be equal to 0 with the increase of the stiffness perturbation. The vibrational energy proportions, as shown in Figures 2c and d, can be used as a quantitative characterization of the level of the mode localization. A “localization factor”^36^ (*γ*) has been used to describe the level of the mode localization.

(10)γ=−lnκ+12∑i=12ln|ωi2−ω02|/ω02=12ln|δκ|

It can be observed from Equation (10) that the level of the mode localization depends only upon the perturbation-to-coupling ratio, and the mode localization becomes more pronounced as this ratio increases^37^. Therefore, with a certain coupling factor, the larger the perturbation is, the deeper the level of mode localization that will be exhibited.

For the DRD scheme, it is reasonable to believe that the even energy distribution point shifts from the usually considered veering point (*δ*=0), and the shifted value is based on the solution of the function $\left|{X_{D1}|}_{\omega_{1}}\right|=\left|{X_{D1}|}_{\omega_{2}}\right|$. According to Equations (3) and (5), and using a numerical solution and linear fitting method, the solution of the equation is *δ*≈−2*κ*. That is, under the DRD scheme, the point where the vibrational energy is evenly distributed in the two vibration modes shifts from the veering point, with a stiffness perturbation of −2*κ* for Resonator 1 and 2*κ* for Resonator 2.

## Experiments

**Figure 3:**
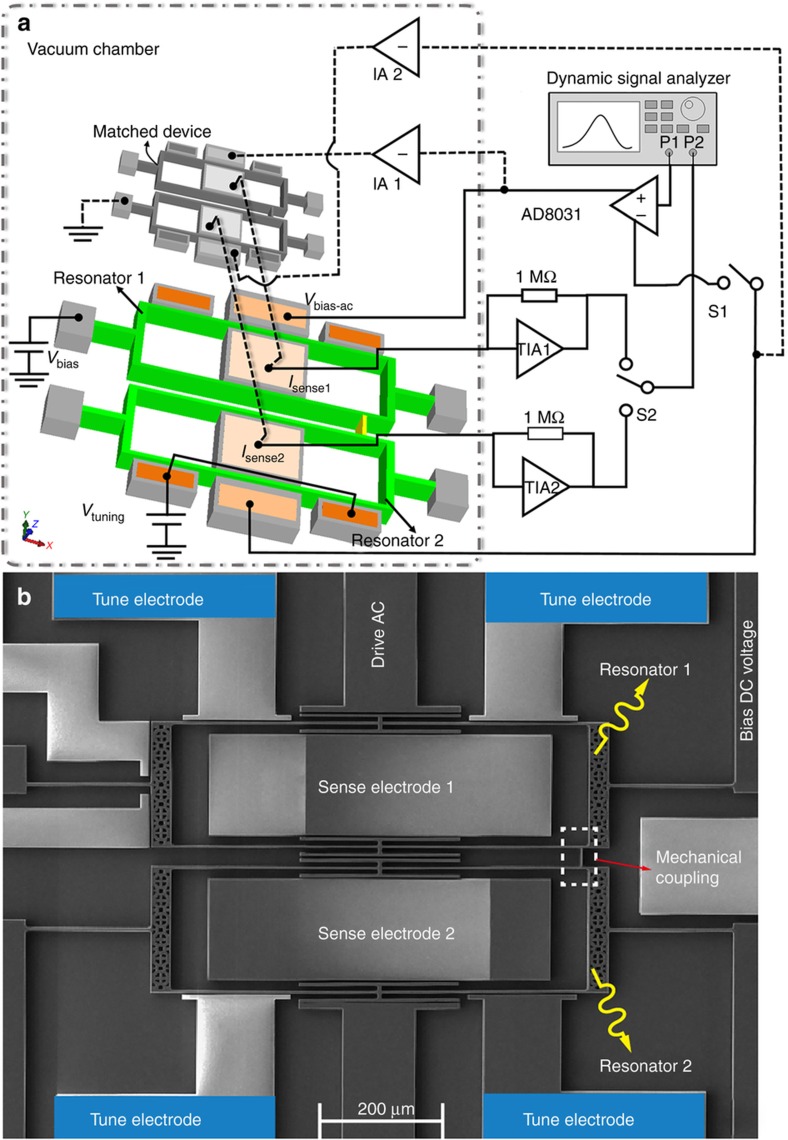
(**a**) Measurement setup with feedthrough cancellation. (**b**) Scanning electron microscope (SEM) image of the device.

To verify the theories experimentally, a 2-DoF WCRs device was designed and fabricated using a silicon-on-insulator (SOI) process^38,39^. The device included two double-ended tuning fork (DETF) resonators coupled by a mechanical beam and four tuning electrodes around the two DETFs that were used to generate artificial stiffness perturbations. Therefore, the device performed as a stiffness sensor. The measurement setup and an SEM image of the device are shown in Figure 3.

The device was actuated and sensed by gap-variation parallel-plate capacitors. The designed actuation and sense capacitances were 27.08 fF and 54.16 fF, respectively. The bias voltage applied to the WCRs was *V*_bias_=25 V. A single-to-differential amplifier (AD8031) was used to generate differential AC sweep signals of equal magnitude that were opposite in phase, such that in-phase forces could be generated. It was the SRD scheme when S1 was open and the DRD scheme when S1 was closed. The motional current was converted to a voltage by a trans-impedance amplifier with a gain of 1 MΩ and measured by a dynamic signal analyzer (Agilent 35670a). The device and measurement circuit were placed in a vacuum chamber with a pressure of ~20 mTorr at room temperature. The stiffness perturbation Δ*k* was generated by adjusting the voltage applied to the four independent tuning electrodes beside Resonator 2. The stiffness perturbation was calculated based on the formula Δ*k*=−*εA·*Δ*V*^2^/*g*^3^, where *ε*=8.85×10^−12^ F m^−1^ is the permittivity of free space, *A*=9600 μm^2^ was the effective cross-sectional area of the tuning capacitors, Δ*V* was the voltage difference between the tuning electrodes and the WCRs, and *g* was the capacitor gap of 3 μm.

### Feedthrough cancellation

Due to the capacitive driving and sensing methods as introduced above, the sensing signal of the stiffness sensor was always accompanied by feedthrough signal. The feedthrough signal was coupled through the driving ports, substrate and package. It was found that the feedthrough signal caused artificial anti-resonance around the resonance frequency^40^ and also influenced the location of the zero point between the two resonances for the WCRs^41^.

To investigate the anti-resonances variation due to the stiffness perturbation but not the feedthrough signal, the feedthrough signal had to be properly eliminated. The feedthrough cancellation method used a matched device with the same structural size as the stiffness sensor but that did not resonate. As shown in Figure 3a, driving signals that were out-of-phase with that added to the stiffness sensor were generated by two inverting amplifiers (IA) and applied to the matched device. The bias port of the matched device was connected to the ground, which meant that the matched device was not resonating; thus amplitude-equivalent and phase-opposite compensation currents were generated. Therefore, by adding the compensation currents to the sensing electrodes of the stiffness sensor, the sensing signals inflowing to the trans-impedance amplifiers became feedthrough-cleaning.

**Figure 4:**
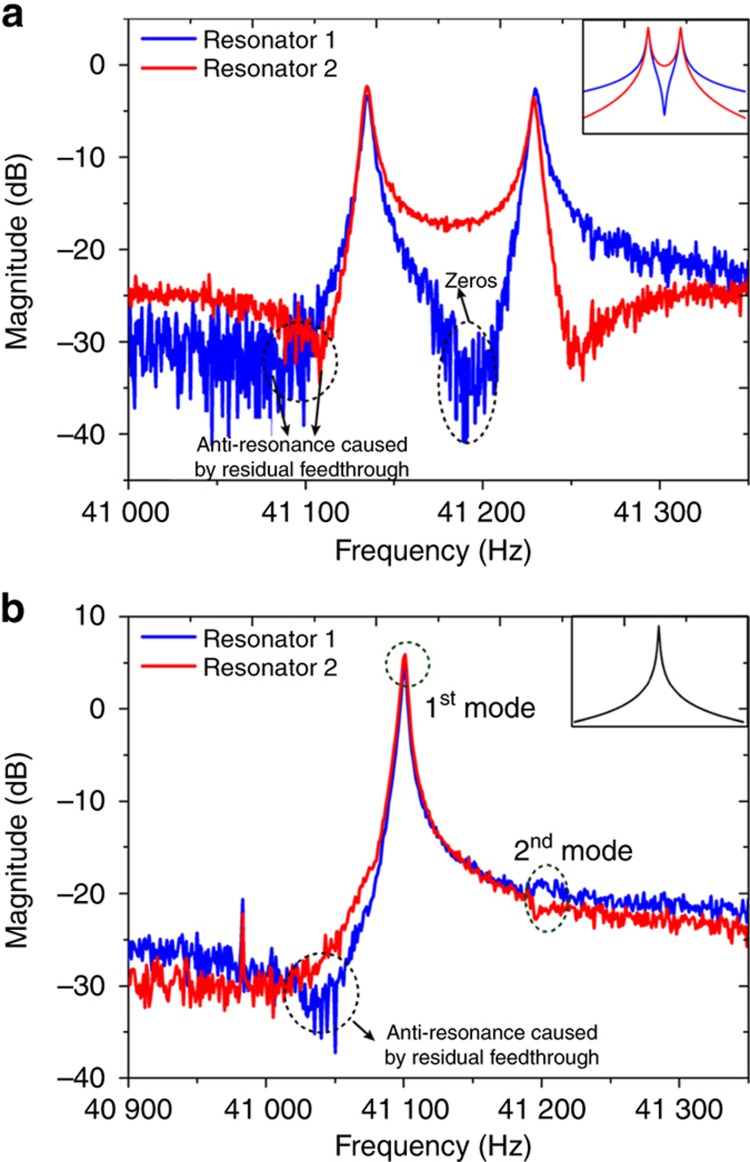
Experimentally measured responses of the two resonators at the veering point under the SRD scheme (**a**) and the DRD scheme (**b**) after feedthrough cancellation. The inset figures indicate the theoretical predictions.

According to theoretical derivations using Equations (2) and (6), the anti-resonance of Resonator 1 under the SRD scheme should be located at the center of the two peaks, *Z*_S1_≈(*ω*_1_+*ω*_2_)/2, without stiffness perturbation (*δ*=0). Figures 4a and b demonstrate the measured responses of the stiffness sensors around the veering point (*δ*=0) after feedthrough cancellation. It can be observed from Figure 4a that the zero point (41185.5 Hz) is very close to the midpoint (41 182.5 Hz) between the first mode (41 133.5 Hz) and second mode (41231.5 Hz), which indicates that the feedthrough signal was almost completely eliminated. However, there was still a small residual feedthrough signal, because the structural sizes and wire bonding between the matched device and the stiffness sensor cannot be completely symmetric. Due to the residual feedthrough signal, the zero point shifted slightly from the theoretically predicted point. In addition, extra light anti-resonances appear at the points with frequencies lower than the first mode.

## Results and discussion

**Figure 5:**
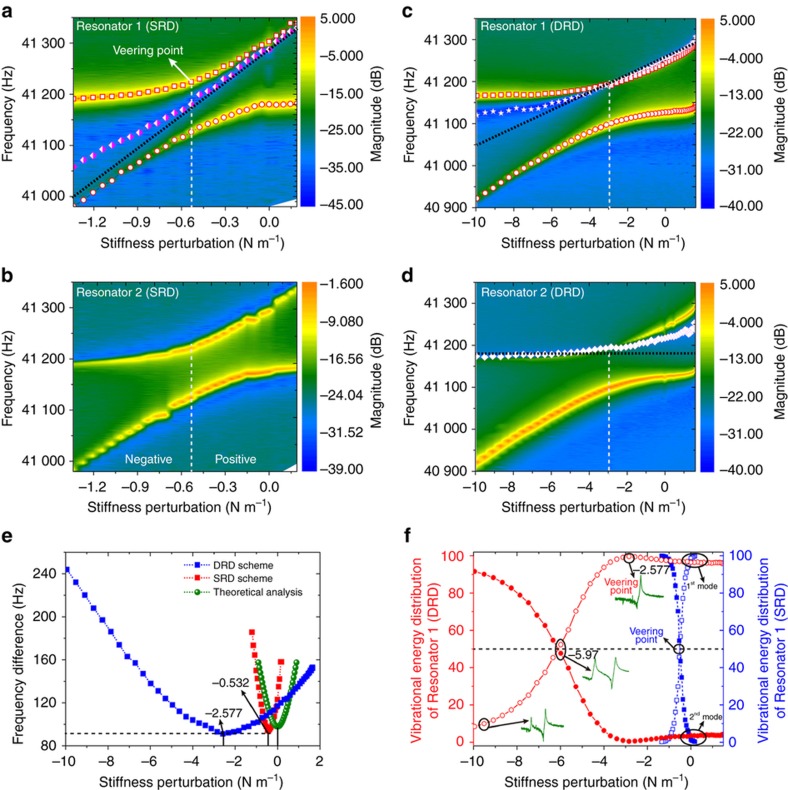
Experimentally measured frequency-perturbation magnitude responses of Resonator 1 (**a** and **c**) and Resonator 2 (**b** and **d**) under the SRD and DRD schemes. The squares show the variations of the resonances of the second mode, and the circles, those of the first mode. The magenta diamonds in (**a**) indicate the variations of the anti-resonances of Resonator 1 under the SRD scheme. The stars in (**c**) and the white diamonds in (**d**) indicate the variations of the anti-resonances of Resonator 1 and Resonator 2 under the DRD scheme, respectively. (**e**) Frequency difference between the two modes under different driving schemes. (**f**) Vibrational energy distributions of Resonator 1 under the two driving schemes. The black dotted lines in (**a**, **c** and **d**) indicate the theoretical anti-resonance loci.

The magnitudes of the frequency responses of the two resonators to stiffness perturbations under the SRD and DRD schemes were recorded, and contour plots in the magnitude-frequency-perturbation plane were obtained. The responses of Resonators 1 and 2 under the SRD scheme are shown in Figures 5a and b, while the responses under the DRD scheme are shown in Figures 5c and d, respectively. The color scale bar indicates the magnitude of the vibration. Figure 5e shows the frequency difference between the two modes, and Figure 5f shows the measured vibrational energy distributions of the two modes of Resonator 1 under the SRD and DRD schemes. In the following, we will discuss the mode localization, anti-resonance behavior, veering point shift, energy proportions and sensitivity comparison of to the results.

We can observe the classical eigenvalue loci veering phenomenon from the two frequency loci under both the SRD (Figure 5a) and DRD schemes (Figure 5c). The resonant frequency lines (squares and circles) all indicate the phenomenon: the resonant frequencies of the two modes repulse each other and do not cross when they approach at the veering point. Considering the vibrational energy, we can observe from Figures 5a and b that the vibrational energies (expressed by the color scale) of both Resonator 1 and Resonator 2 are evenly distributed between the two modes with equal proportions at the veering point. With the variation of the stiffness perturbation, the vibrational energy of Resonator 1 in Figure 5a is no longer evenly distributed between the two modes, while in Figure 5b, the energy distributions in the two modes for Resonator 2 are unchanged. For convenience, the region in which the stiffness perturbation is larger than that of the veering point is termed the positive region and that in which it is smaller is termed the negative region. The vibrational energy of Resonator 1 is predominately confined to the first mode in the positive region, while it is confined to the second mode in the negative region, as indicated by the colored magnitude intensities in Figure 5a. This is the energy confinement phenomenon mentioned above. With the occurrence of energy confinement, we can say that mode localization happens.

The anti-resonance frequencies of Resonator 1 vary almost linearly with the stiffness perturbation under the SRD scheme, as depicted by the diamonds in Figure 5a. The anti-resonance also provides information about the energy confinement, whereas the resonant frequencies cannot. It can be observed that the anti-resonance is close to the center of the two modes when the stiffness perturbation reaches the veering point of Δ*k*=−0.532 N m^−1^. In the positive region, the vibrational energy is predominantly confined to the first mode because the anti-resonance frequencies move closer to the second mode and farther from the first mode. The reverse is true in the negative region, where the vibrational energy is predominantly confined to the second mode because the anti-resonance frequencies move closer to the first mode and farther from the second mode. In summary, the vibrational energy is always confined to the mode farther from the anti-resonances, and the location of the anti-resonance can be used as a signal of the occurrence of energy confinement. In other words, mode localization occurs at a deeper level as the anti-resonance moves closer to the resonance. Therefore, the linear anti-resonance behavior can be regarded as a new manifestation of mode localization in the frequency domain.

However, it can also be observed from Figure 5 that the measured anti-resonance loci are not completely identical to the theoretical prediction. Only half of the anti-resonance loci match the theoretical analysis; that is, the anti-resonance of Resonator 1 in the positive region under both the SRD (Figure 5a) and DRD schemes (Figure 5b). The discrepancies relative to the theoretical prediction are due to the residual feedthrough signal, which induces additional anti-resonances at points with frequencies lower than the first mode and higher than the second.

Under the DRD scheme, for both Resonator 1 and Resonator 2, the anti-resonance loci cross the eigenvalue loci of the second mode at the veering point. The veering point is a special case in which the second mode completely overlaps with the anti-resonance such that the vibrational magnitude of the second mode at the veering point is approximately zero.

From the anti-resonance linear behavior, we know that the location of the anti-resonance is in good agreement with the stiffness perturbation. The anti-resonance should be located at the center of the two modes (resonances) if the two resonators are structurally symmetric, and the anti-resonance frequency will shift from the center with the structural asymmetry of the coupled resonators. In practical applications, by observing the location of the anti-resonance in the frequency responses, we can identify whether the coupled resonators are structurally symmetric.

The frequency difference is another commonly used output metric for model-localized sensors. According to Equation (5), the theoretical frequency difference between the two modes is $\Delta \omega =\omega_{2}-\omega_{1}\approx \omega_{0}\sqrt{\delta^{2}+4\kappa^{2}}/2$. Thus, the theoretical frequency difference for the SRD and DRD can be drawn as the green line in Figure 5e, because the resonant frequency is an inherent property of the resonators and is independent of the driving scheme. It can be observed from Figure 5e that the minimum frequency difference appears at the point of Δ*k*=−0.532 N m^−1^ (*δ*=−0.00355) under the SRD scheme, and Δ*k*=−2.58 N m^−1^ (*δ*=−0.0178) under the DRD scheme, while it should theoretically appear at the veering point, where Δ*k*=0 N m^−1^. The shift of the veering point between the measured SRD line and the theoretical line is caused by the initial structural asymmetry of the weakly coupled resonators due to the fabrication tolerances. Although it is difficult to quantitatively estimate the stiffness and mass mismatches simultaneously, it is possible to use the obtained frequency and veering point information to identify one parameter between them if the other is assumed to be symmetric or has been precisely measured^42^.

The vibrational energy proportions of Resonator 1 under the SRD and DRD schemes are shown in Figure 5f. For the SRD scheme, the proportion of vibrational energy of the first mode is equal to that of the second mode at the veering point of Δ*k*=−0.532 N m^−1^. In contrast, for the DRD scheme, the proportion of vibrational energy of the first mode is ~100% and that of the second mode tends to 0% at the veering point of Δ*k*=−2.58 N m^−1^. The point where the vibrational energy is evenly divided between the two modes appears at Δ*k*=−5.97 N m^−1^ (*δ*=−0.0398) under the DRD scheme. The vibrational energy proportions are nicely consistent with the above theoretical analyses; thus, they provide a quantitative criterion for the level of the mode localization. In practical sensor design, the vibrational energy distributions can be used to determine the upper and lower borders of the measurement range of the mode-localized sensors. Limited by the detection capability of the capacitance sensing method of the MEMS sensors, the amplitude will be difficult to detect if the energy proportion of a particular mode is too small. For example, as shown in Figure 5f under the DRD scheme, the perturbation ranges of Δ*k*>−2.577 N m^−1^ for the second mode and Δ*k*<−10 N m^−1^ for the first mode cannot be used because their vibrational energy proportions are too small (<5%) to be detected. In other words, the linear working range should be limited to [−10, −2.577 N m^−1^] if the first mode is selected as the working mode.

**Figure 6:**
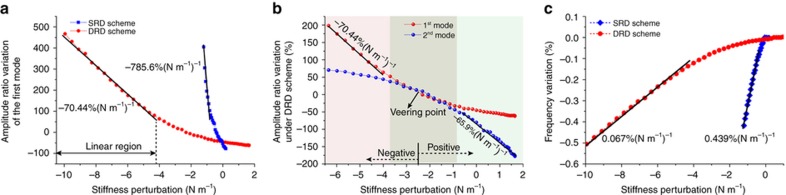
(**a**) Relative variations of the amplitude ratios of the first mode under SRD and DRD schemes. (**b**) Comparison of the amplitude variations of the two modes under DRD schemes. (**c**) Relative variations of the frequency of the first mode under both SRD and DRD schemes.

By calculating the relative variations of the amplitude ratio and frequency with the stiffness perturbation, we found that under the DRD scheme, the amplitude ratio-based sensitivity is approximately an order of magnitude less than that under the SRD scheme. The detailed and accurate relative amplitude ratio variations versus the stiffness perturbations are shown in Figure 6. From the slopes of the curves, we can see that the amplitude ratio sensitivity under the DRD scheme is −70.44% (N m^−1^)^−1^, while that under the SRD scheme is −785.6% (N m^−1^)^−1^. It must be noted that the linear measurement range under the DRD scheme ([−10, −4.4 N m^−1^]) is extended compared to that under the SRD scheme ([−1.2, −0.47 N m^−1^]). Although the sensitivities of the amplitude ratio and frequency under the DRD scheme are not as large as those under the SRD scheme, the range of linear measurement is greatly enhanced. Additionally, the weakly coupled resonators are more easily controlled in the in-phase mode using the DRD scheme in the analog control circuit, because the direction of the excitation forces under the DRD scheme is the same as the in-phase vibrational direction. The DRD scheme is a better choice for sensing applications of weakly coupled micromechanical resonators when a particular mode should be tracked precisely, such as in an accelerometer. In spite of this, the SRD scheme is still very useful in a situation in which no closed-loop control is required, such as in mass sensing. Therefore, different driving schemes can be selected for different applications, depending on the sensitivity and measurement range requirements.

Theoretically, the sensitivity of the first mode in the negative region and sensitivity of the second mode in the positive region should be equivalent, according to the derivation and prediction from Figure 1. It can be observed from Figure 6b that in the symmetry regions with respect to the veering point, that is, [−6.39, −2.57 N m^−1^] in the negative region and [−2.57, 1.61 N m^−1^] in the positive region, the sensitivity of the first mode in the negative region is −70.44% (N m^−1^)^−1^, whereas the sensitivity of the second mode in the positive region is −65.9% (N m^−1^)^−1^. The two measured sensitivities are very close but not completely equivalent, and the inequality is mainly caused by the measurement uncertainty of the amplitude ratio.

## Conclusions

In conclusion, in addition to verifying the two most commonly used characteristics of disordered WCRs, that is, eigenvalue loci veering and energy confinement, two new characteristics of mode localization are discussed.

First, the anti-resonance loci show a linear relationship with respect to the stiffness perturbation. The practical significance of the anti-resonance behavior is that it can characterize the mode localization in the frequency domain and can identify which resonator is perturbed. However, it should be noted that the linear anti-resonance behavior is not very suitable as a sensing mechanism because tracking the anti-resonance introduces larger measurement errors compared to measuring frequency or amplitude shifts. The uncertainty/error of the anti-frequency is related to the phase noise^43^. The signal-to-noise ratio of the anti-resonance is difficult to improve because the amplitude of the anti-resonance is smaller than that of the bias noise.

Second, forced localizations under the SRD and DRD schemes are explored, and the energy distribution trends of the two modes versus the stiffness perturbation are established. The established energy distribution trends can be theoretically used to quantitatively characterize the level of mode localization. In practical mode-localized sensor design, the energy distribution trends can be used to determine the effective working mode and linear measurement range of the sensors. In addition to the well-known sensitivity enhancement of the amplitude ratio readout compared to the frequency readout, we also demonstrated that forced localization under the DRD scheme provides an enhanced linear measurement range while sacrificing a certain degree of sensitivity. The above consequences will contribute to the closed-loop control design, effective linear measurement range determination, and driving of method selection for weakly coupled resonator-based sensors.

However, the design of the WCRs should be improved in future work to avoid the certain structural imbalance caused by tuning the stiffness of the outer tine of the DETF. The locations of the anti-resonances in this work are still somewhat affected by residual feedthrough signals; therefore, the feedthrough cancellation method and the driving/sensing schemes should be optimized to avoid the influence of the electrical parasitic capacitance in future work.
